# Cellphone laws and teens’ calling while driving: analysis of repeated cross-sectional surveys in 2013, 2015, 2017, and 2019

**DOI:** 10.1186/s40621-020-00290-x

**Published:** 2020-12-03

**Authors:** Li Li, Caitlin N. Pope, Rebecca R. Andridge, Julie K. Bower, Guoqing Hu, Motao Zhu

**Affiliations:** 1 Division of Epidemiology, College of Public Health, The Ohio State University, 700 Children’s Drive, Columbus, OH 43205-2664 USA; 2 Graduate Center for Gerontology, Department of Health, Behavior, and Society, College of Public Health, University of Kentucky, 725 Rose Street, Suite 401, Lexington, KY 40536 USA; 3 Division of Biostatistics, College of Public Health, The Ohio State University, 242 Cunz Hall, 1841 Neil Avenue, Columbus, OH 43210 USA; 4 Department of Epidemiology and Health Statistics, Xiangya School of Public Health, Central South University, 110 Xiangya Road, Changsha, 410078 Hunan China; 5 Center for Injury Research and Policy, The Abigail Wexner Research Institute at Nationwide Children’s Hospital, 700 Children’s Drive, Columbus, OH 43205 USA; 6 Department of Pediatrics, College of Medicine, The Ohio State University, 700 Children’s Drive, Columbus, OH 43205-2664 USA

**Keywords:** Distracted driving, Cell phone laws, Handheld ban, Cell phone use, Adolescent

## Abstract

**Background:**

Distracted driving among teens is a public health and safety concern. Most states in the U.S. have sought to restrict cellphone use while driving by enacting laws. This study examines the difference in prevalence of self-reported calling while driving (CWD) between states with different cellphone bans.

**Methods:**

Demographics and CWD data were extracted from state Youth Risk Behavior Surveys (YRBS) from 14 states in 2013, 2015, 2017, and 2019. The state YRBS is conducted every 2 years with a representative sample of 9th through 12th grade students attending public school. States were grouped by type of cellphone law(s): no ban (the absence of both handheld calling ban and young driver ban), young driver ban (a ban on all forms of cellphone use while driving, for young drivers only), or concurrent ban (a young driver ban plus a ban on handheld calling for all drivers irrespective of age). Poisson regression models with robust variance were used to estimate prevalence ratios comparing CWD prevalence across ban types.

**Results:**

In total, 157,423 high school students participated in the surveys, and 65,044 (45%) participants reached the minimum age to obtain an intermediate license and drove during the 30 days prior the survey. Approximately 53% of participants reported CWD at least once during the previous 30 days, and the percentages varied widely by states (range: 51–55%). Compared to students from states with no ban, those from states with concurrent bans were 19%(95% CI: 14–24%) less likely to engage in CWD. Students in states with concurrent bans were 23% less likely to engage in CWD compared to students in states with young driver bans (95% CI:17–27%).

**Conclusions:**

Engaging in CWD is common among teen drivers. The concurrent implementation of a handheld calling ban and a young driver ban was associated with a lower prevalence of CWD.

**Supplementary Information:**

The online version contains supplementary material available at 10.1186/s40621-020-00290-x.

## Background

Drivers aged 16–20 years old are disproportionately at risk for motor vehicle crashes. Compared to drivers of other age groups, teen drivers have the highest crash rate involvement (including fatal crashes, injury crashes, or property-damage-only crashes), even though they accounted for the lowest percentage of licensed drivers on U.S. roadways (Traffic Safety Facts Annual Report Table 62 2019). One common risky driving behavior is engagement in cellphone-related distractions (National Center for Statistics and Analysis 2019). Although reducing cellphone-related distraction is of public health interest for drivers of all ages, surveillance studies have shown persistent engagement over time among teen drivers (Moreno 2013; McCartt et al. 2006; AAA Foundation for Traffic Safety 2018; AAA Foundation for Traffic Safety 2017; Kann et al. 2014; Kann et al. 2016; Kann et al. 2018; Redfield et al. 2020).

Driving simulation and naturalistic driving studies have extensively investigated the negative impact of cellphone use on driving behaviors and outcomes. Consistently, studies have documented evidence of poorer speed maintenance abilities, increased reaction time to hazards, and a higher likelihood of experiencing both near-crashes and crashes for drivers under the influence of distraction (Redelmeier and Tibshirani 1997; Rakauskas et al. 2004; Dula et al. 2011; Caird et al. 2018; Strayer and Johnston 2001; Ishigami and Klein 2009; Choudhary and Velaga 2019; Kass et al. 2007; Gershon et al. 2019). When looking specifically at teen drivers, teens who talked on a cellphone while driving were twice as likely to be involved in property-damage or higher-severity crashes than those who were not talking on a cellphone (Guo et al. 2017).

In response to these traffic safety concerns, U.S. states have enacted various laws to reduce cellphone use while driving, targeting either the mechanism of distraction or the experience-level of the driver. As of March 2020, most U.S. states (except for Montana and Missouri) have enacted texting bans for all drivers, prohibiting manual engagement in text-based communication while driving. Twenty five states and the District of Columbia (D.C.) have also enacted laws to banning handheld mobile phone conversations for drivers of all ages (i.e., handheld calling bans) (Cellphone use laws by state 2019; Ohio Revised Code 2018). Further, 38 states and Washington D.C. have implemented young driver bans, restricting any type of cellphone use while driving for novice drivers (≤ 18 years or drivers with a permit/intermediate driver license) (Cellphone use laws by state 2019).

To assess the effectiveness of these laws, studies have investigated the association between cellphone-related laws and driving outcomes including driver cellphone use and fatal crash rates (McCartt et al. 2006; McCartt et al. 2014). When specifically looking at young drivers, previous research has found that handheld calling bans were related to a 55% reduction of self-reported calling while driving (Rudisill et al. 2018a), a 58% decrease of roadside-observed phone conversations (Zhu et al. 2016), and significant reductions in driver fatalities and the rate of involvement in fatal crashes (Lim and Chi 2013; Rudisill et al. 2018b). When assessing texting bans, studies have reported no significant associations between the enactment of texting bans and the reduction of self-reported engagement in texting (Rudisill et al. 2018a; Rudisill and Zhu 2015), and have seen inconclusive findings with reducing crash fatalities among young drivers (Rudisill et al. 2018b; Ferdinand et al. 2014). Furthermore, studies have found young driver bans lack effectiveness in reducing both short-term (5 months after enactment) and long-term (2 years after enactment) observed cellphone use (Foss et al. 2009; Goodwin et al. 2012). None of these studies assessed the combined effect of a handheld calling ban and a young driver ban in reducing self-reported talking on a phone while driving among teen drivers. To address this research gap in the literature, our study aimed to estimate the association between cellphone laws and the prevalence of talking on a phone while driving among teen drivers by using data from multiple state Youth Risk Behavior Surveys from 2013 to 2019.

## Methods

### Data source and study population

Data was obtained from state Youth Risk Behavior Surveys (YRBSs), which are repeated cross-sectional surveys using a two-stage cluster sample design. State YRBSs are anonymous, voluntary surveys conducted every 2 years to obtain a representative state sample of 9th through 12th grade students attending public school (Kann et al. 2014; Kann et al. 2016; Kann et al. 2018).

The YRBSs sampling design and methodology for combining and analyzing state-level data has been described previously (Kann et al. 2014; Kann et al. 2016; Kann et al. 2018; Youth Risk Behavior Surveillance System 2016). Only state with a response rate ≥ 60% would be weighted and access to public. States that asked a question about talking on a cellphone while driving in at least one of the years 2013, 2015, 2017, or 2019 surveys were included in this analysis. Participating states are listed in Additional File Table 1. The study inclusion criteria were students who had reached their state’s minimum age to obtain an intermediate license and had driven at least once in the 30 days prior to the survey administration date.

Data on state cellphone laws were obtained from the Insurance Institute for Highway Safety (Cellphone use laws by state 2019). Amendments to the laws and their effective dates were identified using the LexisNexis Academic database and state legislative documents (Bill effective dates n.d.). The distribution of enrollment in public elementary and secondary schools by areas were obtained from the National Center for Education Statistics (Percentage distribution of enrollment in public elementary and secondary schools, by school urban-centric 12-category locale and state or jurisdiction 2013). Detailed values of these variables for each state are listed in Additional File Table 2.

### Measures

The study outcome was self-reported talking on a phone while driving (calling while driving, CWD was used as an abbreviation as opposed to TWD because TWD usually refers to texting while driving). CWD, which was measured using the question: “During the past 30 days, on how many days did you talk on a cell phone while driving a car or other vehicle?” Response options included seven ordinal categories ranging from 0 to 30 days. Students who responded “I did not drive” were excluded from the analysis. Analysis using the original seven ordinal categories is in Additional File Table 3. For the descriptive analysis, we categorized responses into never (0 days), sometimes (1–9 days) and frequent (10–30 days) engagement in CWD. For multivariable analysis, we created a binary outcome (never versus at least once) as any exposure to talking on a phone while driving may increase crash risk for teen drivers. A similar binary categorization was utilized by a previously published study using YRBSs data on texting/emailing while driving (Li et al. 2018).

The state status of handheld calling bans and young driver bans were classified as 1) no ban (the absence of both handheld calling ban and young driver ban), 2) young driver ban (a ban on all forms of cellphone use while driving, for young drivers only), or 3) concurrent ban (a young driver ban plus a ban on handheld calling for all drivers irrespective of age), in which all drivers are not allowed to engage in handheld CWD and young drivers cannot engage in any type of cellphone while driving. No state in this study had a handheld calling ban for all drivers without having a young driver ban. Cellphone law information for each state is listed in Additional File Table 2.

Previous studies have reported that teen driver cellphone use, varies by age, sex, race/ethnicity, and urban/rural status (Rudisill et al. 2018a; Rudisill and Zhu 2015; Li et al. 2018; Schroeder et al. 2018; Olsen et al. 2013). We restricted our main analysis to students who had reached the state-dependent age to begin unsupervised driving under certain driving conditions as driving under the supervision of an adult driver may prohibit teen’s CWD behavior (Foss et al. 2009; Graduated licensing laws by state 2020). For our study, urban/rural status was presented by the precent of students in rural areas, calculated by dividing the number of students enrolled in public elementary and secondary schools from rural areas by the total number of students enrolled in public elementary and secondary schools for each state. We used the enrollment from both elementary and secondary schools as data from only secondary schools is not available.

### Statistical analysis

The association of cellphone laws and CWD was examined by adjusting for student demographics, the percent of students in rural areas, and survey year. None of the YBRS’s participating states changed cellphone law status during the study period, therefore, we estimated the difference of CWD between students of states with varing laws, but not the difference of pre-post law periods within states. Crude and adjusted prevalence ratios (PRs) with 95% confidence intervals (CIs) for CWD were estimated using Poisson regression models with robust variances estimation (Barros and Hirakata 2003). Further we included interactions between cellphone laws and student demographics to examine the associations between law types and CWD across the following subgroups, age (15/16 vs. ≥17 years), sex (female or male), race/ethnicity (White, Black or African American, Hispanic/Latino and others).

Complete case analysis was used as the percentage of missing data was low (approximately 2% of students reached the minimum age of intermediate license but did not answer the question on CWD). Data were weighted to adjust for school and student nonresponse, the distribution of students by grade, sex and race/ethnicity, and the complex design (strata and psu) (Kann et al. 2018; Youth Risk Behavior Surveillance System 2016). Data analyses were performed in 2020 using SAS Enterprise Guide 7.1 (SAS Institute Inc., Cary, NC) and STATA 14.0 (StataCorp LLC, College Station, TX).

### Sensitivity analysis

Several sensitivity analyses were conducted to assess potential biases: 1) restricting the analysis to the six states that participated in at least three survey years (Connecticut, Massachusetts, Missouri, Montana, Nebraska, North Dakota); 2) excluding Utah, which enacted their young driver ban during the same year the survey was conducted (2013), thus limiting the sample to states that enacted young driver bans before survey administration; 3) excluding Texas, which was weighted as 39% of the total study population (the total population in Texas is much larger than other participating states; 4) including all students who drove in the past 30 days regardless of their age or licensing status.

Lastly, to estimate the association between cellphone laws and CWD as a nominal outcome, we fitted Poisson regression models to calculate the prevalence ratios for 1) sometimes CWD vs. never CWD, and 2) frequent CWD vs. never CWD.

## Results

In total, 157,423 high school students participated in surveys during 2013, 2015, 2017 and 2019 across 14 states. Approximately 45% (65,044) of total participants reached the minimum intermediate liscense age in their states and reported that they had driven in the past 30 days. Among students who met the inclusion criteria, 53% engaged in CWD at least once during the past 30 days. The prevalence of CWD was highest among older students and lowest for the younger. About 41% of students aged 18 and older sometimes engaged in CWD and 22% of them frequent engaged in CWD, compared to 22% sometimes and 7% frequent among students aged 15 years old. A higher prevalence of White students (60%) at least once engaged in CWD compared to students of other races/ethnicities (42% for Black or African American students, and 45% for Hispanic students). White students had a higher prevalence of engaging in frequent CWD compared to other races. (Table 1, Table 2).

**Table 1:** Characteristics of study population and prevalence of calling while driving (CWD)^a^

Variables	Unweighted N	% of CWD (95%CI)^b^		
		Never (0 day)	Sometimes (1–9 days)	Frequent (10–30 days)
**Overall**	65,044	47 (45, 49)	36 (35, 38)	16 (15, 18)
**Survey year**				
2013	12,166	44 (42, 46)	37 (36, 39)	18 (17, 20)
2015	8717	46 (44, 49)	39 (37, 41)	14 (13, 16)
2017	27,168	50 (47, 53)	36 (33, 38)	15 (13, 17)
2019	16,993	54 (52, 55)	35 (33, 36)	12 (11, 13)
**Age (Years)**				
15	2787	71 (66, 77)	22 (17, 26)	7 (4, 11)
16	26,256	60 (58, 62)	30 (28, 33)	10 (8, 12)
17	25,986	43 (40, 46)	39 (36, 41)	18 (16, 20)
≥ 18	10,015	37 (33, 41)	41 (38, 44)	22 (19, 25)
**Sex**				
Female	31,498	48 (45, 50)	37 (36, 39)	15 (13, 16)
Male	33,230	47 (45, 49)	35 (33, 38)	18 (16, 19)
Missing	316			
**Race**				
White	41,452	40 (38, 42)	40 (38, 42)	20 (19, 22)
Black or African American	7128	58 (53, 63)	30 (25, 34)	12 (9, 15)
Hispanic/Latino	7547	55 (51, 59)	33 (30, 36)	12 (9, 15)
Other^c^	7304	57 (52, 62)	34 (28, 39)	9 (7, 12)
Missing	1613			
**States**				
AK	1262	54 (50, 58)	35 (31, 39)	11 (8, 13)
AR	907	37 (33, 42)	37 (33, 41)	26 (22, 30)
CT	3717	62 (60, 64)	29 (27, 31)	9 (8, 10)
MD	30,657	67 (66, 68)	25 (24, 26)	8 (7, 8)
MA	4201	54 (50, 58)	35 (32, 37)	12 (10, 13)
MO	2647	39 (36, 41)	42 (39, 45)	19 (18, 21)
MT	10,292	39 (38, 41)	43 (42, 44)	17 (16, 19)
NE	2325	33 (30, 35)	49 (46, 51)	19 (16, 21)
NJ	617	46 (40, 52)	37 (33, 42)	16 (12, 21)
ND	4606	28 (26, 30)	49 (47, 51)	23 (21, 24)
RI	859	49 (46, 53)	40 (34, 46)	11 (7, 14)
SC	1007	46 (42, 51)	33 (29, 38)	20 (17, 23)
TX	864	49 (44, 54)	35 (31, 39)	16 (13, 19)
UT	1083	35 (30, 41)	48 (43, 52)	17 (15, 19)

*CI* Confidence Interval;

^a^Data were from state Youth Risk Behavior Surveys in 14 states (2013, 2015, 2017 and 2019), the United States.

^b^Weighted percentage of students that reported talking on a phone while driving during the 30 days before the survey (among students who drove). Percentages may not total 100 due to rounding.

^c^Other included: American Indian/Alaskan Native, Asian, Native Hawaiian or Other Pacific Islander, and Multiple- Non-Hispanic/Latino

**Table 2:** Associations between cellphone laws and calling while driving (CWD)^a^

Variables	CWD ^**b**^ (95%CI)	Prevalence Ratios (95%CI)	
		Crude	Adjusted^**c**^
**Overall**	**53 (51, 55)**		
**Cellphone laws**			
No ban	57 (55, 60)	Reference	Reference
Young driver ban	54 (51, 57)	0.94 (0.87, 1.00)	1.05 (1.00, 1.10)
Concurrent bans^d^	44 (41, 47)	0.76 (0.71, 0.83)	**0.81 (0.76, 0.86)**
**Age (Years)**			
15	29 (23, 34)	**0.72 (0.59, 0.88)**	**0.65 (0.56, 0.75)**
16	40 (38, 42)	Reference	Reference
17	57 (54, 60)	**1.42 (1.32, 1.52)**	**1.54 (1.47, 1.60)**
≥ 18	63 (59, 67)	**1.57 (1.46, 1.70)**	**1.68 (1.61, 1.77)**
**Sex**			
Female	52 (50, 55)	Reference	Reference
Male	53 (51, 55)	1.02 (0.96, 1.07)	1.01 (0.97, 1.04)
**Race**			
White	60 (58, 62)	Reference	Reference
Black or African American	42 (37, 47)	**0.70 (0.63, 0.77)**	**0.67 (0.61, 0.74)**
Hispanic/Latino	45 (41, 49)	**0.75 (0.70, 0.81)**	**0.77 (0.72, 0.82)**
Other^e^	43 (38, 48)	**0.71 (0.64, 0.79)**	**0.74 (0.69, 0.80)**
**Percent of students in rural areas**		**1.01 (1.01, 1.01)**	**1.01 (1.01, 1.01)**
**Year**			
2013	57 (54, 59)	Reference	Reference
2015	54 (52, 57)	**0.93 (0.88, 0.97)**	**0.93 (0.88, 0.97)**
2017	50 (47, 54)	0.94 (0.89, 1.01)	0.94 (0.89, 1.01)
2019	44 (43, 46)	0.95 (0.90, 1.01)	0.95 (0.90, 1.01)

*CI* Confidence Interval;

^a^Data were from state Youth Risk Behavior Surveys in 14 states (2013, 2015, 2017 and 2019), the United States.

^b^Weighted percentage of calling while driving (CWD): Percentage of students that reported talking on a phone while driving at least once during the 30 days before the survey (among students who drove).

^c^Model adjusted for cellphone laws, age, sex, race, percent of students in rural area, and survey year.

^d^Concurrent bans: both a handheld calling ban and a young driver ban.

^e^Other included: American Indian/Alaskan Native, Asian, Native Hawaiian or Other Pacific Islander, and Multiple- Non-Hispanic/Latino

**Fig. 1:**
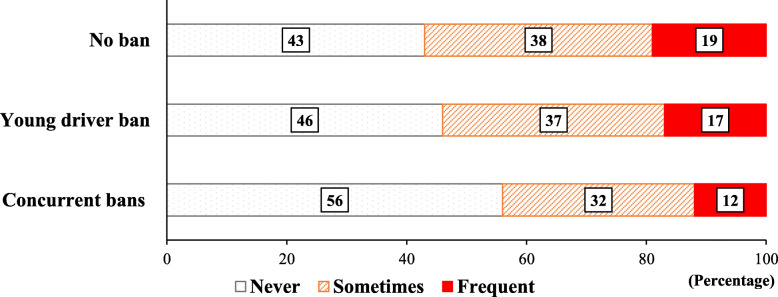
Percentage of teens’ calling while driving by cellphone laws. Notes: a. Data were from state Youth Risk Behavior Surveys in 14 states (2013, 2015, 2017 and 2019), the United States; b. Weighted percentage of calling while driving: Percentage of students that reported talking on a phone while driving at least once during the 30 days before the survey (among students who drove); c. Concurrent bans: both a handheld calling ban and a young driver ban

CWD prevalence varied across states, from 33% in Maryland to 72% in North Dakota (Table 1). States with no ban had a higher percentage of students who sometimes or frequent engaged in CWD (57%) compared to states with concurrent bans (44%) (Fig. 1). Students in states with a young driver ban had a lower prevalence of CWD compared to states with no ban (54% vs 57%), though this difference was not statistically significant in the adjusted model. (Table 2).

Multivariable analysis showed that students in states with concurrent bans were 19% less likely to report CWD compared to students in states with no ban (adjusted PR = 0.81, 95% CI: 0.76–0.86) (Table 2). Similarly, students in states with concurrent bans were 23% less likely to engage in CWD compared to students in states with only a young driver ban. (adjusted PR = 0.77, 95% CI: 0.73–0.83).

The association between law and CWD stratified by subgroups were presented in Table 3. Adjusted PRs by demographics were similar to the main analysis without interactions. Young driver ban was not associated with a lower prevalence of CWD across subgroups. Black/African American or Hispanic/Latino students who in states with a young driver ban had a slightly, but not statistically significant, lower prevalence of CWD. The association between concurrent bans and CWD was stronger among younger drivers (15 and 16 years) (adjusted PR = 0.54, 95% CI: 0.49–0.60), or those of Hispanic/Latino race (adjusted PR = 0.66, 95% CI: 0.57–0.77) compared to the estimation without interaction (adjusted PR = 0.81, 95% CI: 0.76–0.86).

**Table 3:** Adjusted prevalence ratios and 95%CI stratified by student demographics

Variables	Young driver ban	Concurrent bans	No ban	*P*-values^&^
Age^#^				< 0.0001
15–16 years	1.02 (0.94, 1.11)	**0.54 (0.49, 0.60)**	Reference	
≥ 17 years	**1.08 (1.03, 1.14)**	**0.90 (0.84, 0.96)**	Reference	
Sex^$^				0.0151
Female	1.02 (0.96, 1.08)	**0.76 (0.70, 0.82)**	Reference	
Male	**1.07 (1.01, 1.14)**	**0.86 (0.80, 0.93)**	Reference	
Race^*^				0.0229
White	**1.06 (1.01, 1.10)**	**0.83 (0.78, 0.89)**	Reference	
Black or African American	0.97 (0.77, 1.22)	**0.74 (0.62, 0.89)**	Reference	
Hispanic/Latino	0.93 (0.82, 1.06)	**0.66 (0.57, 0.77)**	Reference	
Other^a^	1.08 (0.92, 1.26)	0.89 (0.78, 1.02)	Reference	

*CI* Confidence Interval;

^&^*p*-values for the interaction terms of age*law, sex*law, and race*law.

^#^model adjusted for age, and interaction between age and cellphone laws, sex, race, the state’s percent of students in rural areas, and survey year.

^$^model adjusted for sex, and interaction between sex and cellphone laws, age, race, the state’s percent of students in rural areas, and survey year.

*model adjusted for race, and interaction between race and cellphone laws, age, sex, the state’s percent of students in rural areas, and survey year.

^a^Other included: American Indian/Alaskan Native, Asian, Native Hawaiian or Other Pacific Islander, and Multiple- Non-Hispanic/Latino

Results of the sensitivity analyses were similar to the main analysis (Additional File Table 4 and Table 5). When categorizing CWD as a three-level nominal outcome, students in states with concurrent bans had a lower risk of sometimes engaging in CWD compared to students in states with no ban (adjusted PR = 0.80, 95% CI: 0.74–0.87). Additionally, concurrent bans were associated with a 30% lower prevalence of frequent CWD compared to no bans. (adjusted PR =0.70, 95% CI: 0.60–0.80) (Additional File Table 5).

## Discussion

This study is the first to assess the combined effect of two types of state cellphone law (concurrently had a handheld calling ban and a young driver ban) on teens’ self-reported CWD. We found that over half of teen drivers engaged in CWD at least once during the 30 days prior to their survey response. Compared to states with no bans, the prevalence of CWD was 19% lower in states with concurrent bans.

Our findings regarding the association between the presence of a handheld calling ban and teen’s CWD support and extend previous findings. Rudisill et al., found the percentage of adolescent drivers (16–18 years) engaging in self-reported CWD was lower in states with a handheld calling ban compared to states without a ban, by using self-reported data from 2011 to 2014 (Rudisill et al. 2018a). The association was stronger than that reported in our study, PR = 0.45 (95% CI: 0.32–0.63) versus PR = 0.81 (95% CI: 0.76–0.86). Our study was a cross-sectional design that compared students from states with and without a cellphone law, while Rudisill’s study included both the difference between and within states. The discrepancies between the two studies may also be from differences between selected study populations. For example, our study population was comprised of drivers reaching the minimum age of intermediate license from public high schools in 14 states, whereas the study population in Rudisill et al., was comprised of adolescents aged ≥16 years from household samples where the parent was affiliated with an online probability-based research panel across the U.S.

We found a strong association between concurrent bans and CWD among students aged 15/16 (PR = 0.54, 95%CI: 0.49–0.60) compared to older students. This may be because those students are more likely to hold an intermediate license rather than a full license, and in turn are more likely to drive with an adult in the car. This adult driver would also be impacted by the concurrent ban and may influence the younger driver.

Black or Hispanic/Latino drivers in states with a young driver ban had a lower prevalence of CWD compared to those in states without a young drive ban, though these associations were not statistically significant. Further investigation with longitudinal data are needed to confirm with those findings.

Similar to our primary anlaysis, there is no association between the presence of young driver ban and CWD by age or sex. One possible explanation for the lack of an association between young driver bans and CWD may involve the level of awareness teen drivers possess regarding cellphone laws. A survey conducted in North Carolina found less than two-thirds of teens were aware of the cellphone restriction in their state as far as 2 years after the implementation of the young driver ban (Goodwin et al. 2012). Another issue is the challenge of enforcing cellphone laws. Analysis of citation data from 14 states and D.C. has found that overall enforcement of cellphone bans was low, with cellphone-related distracted driving citations comprising only 1% of all written citations (Rudisill and Zhu 2016). Young driver ban violations accounted for only 2.7/1000 of all teen traffic citations, less than handheld violations for young drivers (9.6/1000) (Rudisill and Zhu 2016). Qualitative research has also shown that police officers have a sense of discomfort in ticketing for cellphone-related distracted driving. As it can be ambiguous what drivers are actually doing with their phone while driving, along with the low rate of admittance from drivers on their engagement in distracted driving (Rudisill et al. 2019; Nevin et al. 2017).

Along with the effectiveness of cellphone bans, cultural and environmental factors have critical roles in shaping young drivers’ behavior (Gershon et al. 2017; Atchley et al. 2012) As driving is a learned behavior, parents/guardians serve as the primary role models for teen drivers and contribute to their overall traffic safety culture (Gershon et al. 2017; Hartos et al. 2002; Raymond Bingham et al. 2015). Survey results from Carter et al. reported that teens with parents who engaged in distracted driving had a higher percent of engaging in distraction tasks (Carter et al. 2014). In states with a young driver ban only, teen passengers may be more likely to observe their parents and other adults engaging in CWD. This could potentially send a mixed message that once driving is “mastered,” engagement in CWD is safe. On the other hand, in states with handheld calling bans, irrespective of driver age, adults were less likely to engage in CWD (McCartt et al. 2010), meaning teen drivers would potentially be less likely to be exposed to adult drivers engaging in cellphone-related distracted driving.

From a clinical and policy perspective, these findings, in combination with previous findings on cellphone bans provide further support for the utility of handheld bans for drivers of all ages. Pediatricians should routinely discuss avoidance of distracted driving with teens during yearly physical exams. Promotion of safer alternatives such as hands-free options or technology which blocks cellular use while driving (e.g. Do Not Disturb mode), should be recommended as motor vehicle crashes remain a leading cause of injury and death for this age group. Although young driver bans target a population of vulnerable road users, legislative efforts that acknowledge unsafe driving behavior irrespective of age will promote a safer driving culture and will translate into safer roads for all drivers.

### Limitations

There are several limitations in our study. First, the questionnaire was framed to inquire about CWD, though information was not available to differentiate handheld or hands-free calling behavior. Self-reported data showed that the enactment of a handheld cellphone ban reduced overall handheld cellphone use while driving, but increased the use of hands-free technology (Carpenter and Nguyen 2015; Bratiman and McCartt 2010). Therefore, our analysis may underestimate the effect of a handheld calling ban as drivers in states with the ban may switch to hands-free technology to avoid a ticket. Second, since this was a cross-sectional study, the analysis cannot imply causality of legislation on drivers’ behavior. Therefore, the association we found between concurrent laws and CWD could be due to unmeasured confounders across states.

Third, few states adapted the question on CWD for their YRBS, restricting the analysis to 14 states with only six states having data for at least 3 years. The difference in CWD across law types may also be attributed to differences between states. It is ideal to fit a multilevel Poisson model with states as random intercepts, but YRBS data only provides a variable for final weights and there are no weights available for each level. In an effort to account for this potential bias, we conducted several sensitivity analyses which yielded similar results between law type and the prevalence of CWD. Fourth, as none of the participating states had a handheld calling ban without a young driver ban, we cannot estimate the association of a handheld calling ban versus no ban, or directly compare the handheld calling ban versus a young driver ban. However, the prevalence ratio between concurrent bans versus no ban was similar to the prevalence ratio between concurrent bans versus a young driver ban, and the young driver ban was non-statistically associated with a reduction in CWD. It is reasonable to believe that a handheld calling ban alone would also be associated with a reduction in CWD, but further investigation is needed.

## Conclusions

Overall, teens in states with concurrent bans (both handheld calling ban and young driver ban) had a lower prevalence of CWD. While the overall effectiveness of cellphone laws need further investigation, it is apparent that restricting drivers of all ages (handheld calling ban), including teens, may influence the traffic safety culture on distracted driving.

## Supplementary Information

**Additional file 1.**

## Data Availability

The datasets generated and analyzed during the current study are available in the CDC Youth Risk Behavior Surveillance System website. https://www.cdc.gov/healthyyouth/data/yrbs/data.htm

## Abbreviations

CWD: Calling while driving (talking on a phone while driving for this study)
CI: Confidence interval
D.C.: District of Columbia
PR: Prevalence ratio
U.S.: United States
YRBS: Youth Risk Behavior Survey
