# Supplementary material for: Cellphone laws and teens’ calling while driving: analysis of repeated cross-sectional surveys in 2013, 2015, 2017, and 2019
Source: Inj Epidemiol. 2020 Dec 3;7:65. doi: 10.1186/s40621-020-00290-x (PMC7713022; doi:10.1186/s40621-020-00290-x)
Supplement: Supplementary file 1 — Additional file 1. [file 40621_2020_290_MOESM1_ESM.docx]

**Additional Files:**

**Additional File Table 1: States included in the study**

| **State** | **2013** | **2015** | **2017** | **2019** |
| --- | --- | --- | --- | --- |
| Alaska | -- | -- | √ | √ |
| Arkansas | √ | -- | -- | -- |
| Connecticut | √ | √ | √ | √ |
| Maryland | -- | -- | √ | √ |
| Massachusetts | √ | √ | √ | -- |
| Missouri | √ | √ | √ | -- |
| Montana | √ | √ | √ | -- |
| North Dakota | √ | √ | √ | √ |
| Nebraska | √ | √ | -- | √ |
| New Jersey | √ | -- | -- | -- |
| Rhode Island | -- | -- | √ | -- |
| South Carolina | √ | -- | -- | -- |
| Texas | -- | -- | √ | -- |
| Utah | √ | -- | -- | -- |

Notes:

a. Data were from state Youth Risk Behavior Surveys in 2013, 2015, 2017 and 2019, the United States;

b. “√”: participated the survey and included the question about talking on a phone while driving (CWD);

c. “--”: Did not participate the survey or did not include the question about CWD.

**Additional File Table 2: Cellphone laws and state-level covariates**

| **State** | **Young driver ban**  **(Effective year)** | **Handheld calling ban**  **(Effective year)** | **Percent of students enrolled in rural** |
| --- | --- | --- | --- |
| **Alaska** | No ban | No ban | 29.4 |
| **Arkansas** | Yes, 2009 | No ban | 33.7 |
| **Connecticut** | Yes, 2005 | Yes, 2005; Not allow to talk into or listen on a hand-held mobile telephone | 12.1 |
| **Massachusetts** | Yes, 2010 | No ban | 9.3 |
| **Maryland** | Yes, 2005 | Yes, 2010; Not allow to use the driver's hands to use a handheld telephone other than to initiate or terminate a wireless telephone call or to turn on or turn off the handheld telephone | 13.9 |
| **Missouri** | No ban (only ban texting for young drivers, 2009) | No ban | 26.4 |
| **Montana** | No ban | No ban | 36.0 |
| **Nebraska** | Yes, 2008 | No ban | 24.9 |
| **North Dakota** | Yes, 2012 | No ban | 40.0 |
| **New Jersey** | Yes, 2002 | Yes, 2004; Not allow to talk or listen to another person on the handheld telephone | 8.1 |
| **Rhode Island** | Yes, 2006 | No ban during study period (Effective in 2018) | 10.2 |
| **South Carolina** | No ban | No ban | 32.9 |
| **Texas** | Yes, 2009 | No ban | 15.7 |
| **Utah** | Yes, 05/14/2013 | No ban | 10.4 |

Note:

a. Missouri enacted a ban in 2009 to ban texting while driving for drivers under 21 years. It did not meet the definition of young driver ban in this study (ban any type of cellphone use among young drivers);

b. Source: Insurance Institute for Highway Safety and National Center for Education Statistics.

**Additional File Table 3 Prevalence and 95%CI of calling while driving using the original seven categories**

| **Variables** | **Never** | **1 or 2 days** | **3 to 5 days** | **6 to 9 days** | **10 to 19 days** | **20 to 29 days** | **All 30 days** |
| --- | --- | --- | --- | --- | --- | --- | --- |
| **Overall** | 47(45,49) | 18(17,20) | 11(10,11) | 7(6,8) | 6(5,7) | 4(3,4) | 6(5,7) |
| **Survey year** |  |  |  |  |  |  |  |
| 2013 | 44(42,46) | 19(18,20) | 11(10,12) | 8(7,9) | 8(7,9) | 4(4,5) | 6(5,7) |
| 2015 | 46(44,49) | 19(17,20) | 12(11,13) | 9(7,10) | 6(6,7) | 4(3,5) | 4(3,5) |
| 2017 | 50(47,53) | 19(17,20) | 10(9,11) | 7(6,8) | 5(4,7) | 3(3,4) | 6(5,7) |
| 2019 | 54(52,55) | 19(18,20) | 9(8,10) | 6(5,7) | 5(5,6) | 3(2,3) | 4(3,5) |
| **Age (Years)** |  |  |  |  |  |  |  |
| 15 | 71(66,77) | 15(12,19) | 4(2,6) | 2(1,3) | 3(1,6) | 0.4(0.02,0.8) | 3(1,6) |
| 16 | 60(58,62) | 17(16,19) | 7(6,9) | 5(4,7) | 4(3,5) | 2(1,3) | 4(2,5) |
| 17 | 43(40,46) | 19(17,21) | 12(10,13) | 8(7,9) | 7(6,8) | 4(3,5) | 7(5,9) |
| ≥18 | 37(33,41) | 19(16,21) | 13(11,15) | 9(7,11) | 8(6,9) | 5(4,6) | 9(7,11) |
| **Sex** |  |  |  |  |  |  |  |
| Female | 48(45,50) | 19(18,20) | 11(10,13) | 7(6,8) | 6(5,7) | 4(3,4) | 5(4,7) |
| Male | 47(45,49) | 18(16,20) | 10(8,11) | 8(6,9) | 7(6,7) | 4(3,4) | 7(6,9) |
| **Race** |  |  |  |  |  |  |  |
| White | 40(38,42) | 20(19,21) | 11(10,13) | 9(8,10) | 8(7,9) | 5(4,6) | 7(6,8) |
| Black or African American | 58(53,63) | 16(12,20) | 9(7,11) | 5(3,7) | 4(2,5) | 1.2(0.7,1.7) | 7(4,10) |
| Hispanic/Latino | 55(51,59) | 17(16,19) | 10(9,12) | 5(4,7) | 5(3,7) | 2(1,3) | 5(3,7) |
| Other ^c^ | 57(52,62) | 17(13,20) | 8(6,11) | 8(3,13) | 3(2,4) | 2(1,4) | 4(3,6) |
| **States** |  |  |  |  |  |  |  |
| AK | 54(50,58) | 18(15,21) | 10(7,13) | 7(5,8) | 5(4,6) | 2(1,3) | 4(1,6) |
| AR | 37(33,42) | 16(12,20) | 11(9,13) | 9(7,11) | 10(8,11) | 4(3,6) | 12(9,15) |
| CT | 62(60,64) | 16(15,18) | 9(7,10) | 4(3,5) | 4(4,5) | 1.4(1.1,1.8) | 3(3,4) |
| MD | 67(66,68) | 15(14,16) | 7(6,7) | 4(3,4) | 3(3,3) | 1.4(1.2,1.6) | 3(3,4) |
| MA | 54(50,58) | 18(16,19) | 9(8,11) | 7(6,9) | 5(4,6) | 3(2,3) | 3(3,4) |
| MO | 39(36,41) | 20(18,22) | 13(11,14) | 10(8,11) | 8(8,9) | 5(4,6) | 6(4,7) |
| MT | 39(38,41) | 22(21,23) | 13(12,13) | 9(8,9) | 9(8,10) | 4(4,5) | 4(4,5) |
| NE | 33(30,35) | 25(23,27) | 14(12,15) | 10(9,12) | 9(7,10) | 5(4,6) | 5(4,6) |
| NJ | 46(40,52) | 19(16,23) | 11(8,13) | 7(5,9) | 8(5,11) | 3(1,5) | 5(3,8) |
| ND | 28(26,30) | 23(21,25) | 14(13,16) | 11(10,12) | 11(10,12) | 7(6,8) | 5(4,6) |
| RI | 49(46,53) | 22(17,28) | 9(8,11) | 8(6,10) | 4(2,6) | 2(1,3) | 5(2,7) |
| SC | 46(42,51) | 16(14,19) | 10(8,12) | 7(5,9) | 7(5,8) | 4(3,6) | 9(6,11) |
| TX | 49(44,54) | 18(15,21) | 10(8,12) | 7(4,9) | 5(3,7) | 4(3,5) | 8(5,10) |
| UT | 35(30,41) | 23(20,26) | 14(11,17) | 11(9,12) | 8(6,10) | 4(3,6) | 5(3,6) |

Notes:

CI: Confidence Interval;

a: Data were from state Youth Risk Behavior Surveys in 14 states (2013, 2015, 2017 and 2019), the United States;

b. Weighted percentage of students that reported talking on a phone while driving during the 30 days before the survey (among students who drove). Percentages may not total 100 due to rounding;

c: Other included: American Indian/Alaskan Native, Asian, Native Hawaiian or Other Pacific Islander, and Multiple- Non-Hispanic/Latino.

**Additional File Table 4: Sensitivity analysis with subset: prevalence ratios** ^a^

| **Cellphone laws** | **Six states** ^b^ | **UT excluded** ^c^ | **TX excluded** ^d^ | **All driver** ^f^ | **Main analysis** |
| --- | --- | --- | --- | --- | --- |
| **No. of participants** | **27,788** | **63,961** | **64,180** | **81,929** | **65,044** |
| **Young driver ban vs. no ban** | 1.01(0.96, 1.05) | 1.04(0.99, 1.09) | 1.02(0.98, 1.06) | **1.08(1.03, 1.13)** | 1.05 (1.00, 1.10) |
| **Concurrent bans** ^g^ **vs. no ban** | **0.78(0.73, 0.84)** | **0.83(0.78, 0.89)** | **0.81(0.76, 0.87)** | **0.81(0.76, 0.87)** | **0.81 (0.76, 0.86)** |
| **Concurrent bans vs. Young driver ban** | **0.78(0.73, 0.84)** | **0.80(0.75, 0.85)** | **0.79(0.75, 0.84)** | **0.75(0.71, 0.80)** | **0.77 (0.73, 0.83)** |

Notes:

a. Data were from state Youth Risk Behavior Surveys in 14 states (2013, 2015, 2017, and 2019), the United States;

b. Seven states: restricted analysis in the five states that participated in at least three of the 4 survey years (CT, MA, MO, MT, ND, and NE);

c. Utah excluded: since the state enacted young driver ban in 2013, the same year as the survey was conducted; All other states enacted young driver bans before 2013;

d. Texas excluded: it weighted as 39% of the total study population;

f. All driver: included all students who reported driving in the past 30 days;

g. Concurrent bans: enacted both a handheld calling ban and a young driver ban;

h. The outcome was the prevalence of talking on a phone while driving at least once in the 30 days before the survey. Models adjusted for age, sex, race, the state’s percent of students in rural areas, and survey year.

**Additional File Table 5: Sensitivity analysis with multinomial outcome: prevalence ratios**

| **Cellphone laws** | **Sometimes vs Never** | **Frequent vs Never** |
| --- | --- | --- |
| **# of participants** | **55,159** | **43,932** |
| **Young driver ban vs. no ban** | 1.06(0.99, 1.12) | 1.10(1.00, 1.21) |
| **Concurrent bans vs. no ban** | **0.80(0.74, 0.87)** | **0.70(0.60, 0.80)** |
| **Concurrent bans vs. Young driver ban** | **0.76(0.70, 0.82)** | **0.63(0.55, 0.73)** |

Notes:

a. Data were from state Youth Risk Behavior Surveys in 14 states (2013, 2015, 2017 and 2019), the United States;

b. Sometimes: engaged in talking on a phone while driving (CWD) “1-9 days” during the past 30 days;

c. Frequent: engaged in talking on a phone while driving (CWD) “10-30 days” during the past 30 days;

d. Concurrent bans: enacted both a handheld calling ban and a young driver ban;

e. Models adjusted for age, sex, race, the state’s percent of students in rural areas, and survey year
